# SRD5A3-CDG: Emerging Phenotypic Features of an Ultrarare CDG Subtype

**DOI:** 10.3389/fgene.2021.737094

**Published:** 2021-12-01

**Authors:** Nazreen Kamarus Jaman, Preeya Rehsi, Robert H. Henderson, Ulrike Löbel, Kshitij Mankad, Stephanie Grunewald

**Affiliations:** ^1^ Metabolic Department, Great Ormond Street Hospital NHS Foundation Trust, London, United Kingdom; ^2^ Ophthalmology Department, Great Ormond Street Hospital, London, United Kingdom; ^3^ Ophthalmology Department, Moorfields Eye Hospital, London, United Kingdom; ^4^ Department of Radiology, Great Ormond Street Hospital for Children, London, United Kingdom; ^5^ Institute for Child Health, NIHR Biomedical Research Center (BRC), University College London, London, United Kingdom

**Keywords:** SRD5A3-CDG, steroid 5 alpha reductase deficiency, emerging phenotypic features, retinal dystrophy, congenital disorder of glycosylation, CDG

## Abstract

**Background:** SRD5A3-CDG is a rare N-glycosylation defect caused by steroid 5 alpha reductase type 3 deficiency. Its key feature is an early severe visual impairment with variable ocular anomalies often leading to diagnosis. Additional symptoms are still poorly defined. In this case study, we discuss 11 genetically confirmed cases, and report on emerging features involving other systems in addition to the eye phenotype.

**Methods:** In total, 11 SRD5A3-CDG patients in five sets of sibships were included in the study. Data on 9 of 11 patients are as of yet unpublished. Patients’ results on biochemical and genetic investigations and on in-depth phenotyping are presented.

**Results:** Key diagnostic features of SRD5A3-CDG are ophthalmological abnormalities with early-onset retinal dystrophy and optic nerve hypoplasia. SRD5A3-CDG is also characterized by variable neurological symptoms including intellectual disability, ataxia, and hypotonia. Furthermore, ichthyosiform skin lesions, joint laxity, and scoliosis have been observed in our cohort. We also report additional findings including dystonia, anxiety disorder, gastrointestinal symptoms, and MRI findings of small basal ganglia and mal-rotated hippocampus, whereas previous publications described dysmorphic features as a common finding in SRD5A3, which could not be confirmed in our patient cohort.

**Conclusion:** The detailed description of the phenotype of this large cohort of patients with SRD5A3-CDG highlights that the key clinical diagnostic features of SRD5A3-CDG are an early onset form of ophthalmological problems in patients with a multisystem disorder with variable symptoms evolving over time. This should aid earlier diagnosis and confirms the need for long-time follow-up of patients.

## Introduction

Congenital disorders of glycosylation (CDG) are genetic diseases with an extremely broad spectrum of clinical presentation. They occur due to defective glycosylation of glycoproteins or glycolipids, and the genetic defects affect several glycosylation pathways (Ondruskova et al., 2021). Initial steps in the glycosylation pathways result in a stepwise build-up of monosaccharide and oligosaccharide chains. Dolichol acts as a carrier of the oligosaccharide precursor during the assembly, occuring initially at the outside and, subsequently, at the inside of the endoplasmic reticulum (ER) membrane. Dolichol biosynthesis defects lead to a congenital disorder of glycosylation. The first step in the dolichol synthesis is dehydrodolichyl diphosphate synthase (DHDDS), which forms a complex with the nuclear undecaprenyl pyrophosphate synthase 1 (NUS), forming polyprenol pyrosphosphate from farnesyl and isoprenenyl diphosphate. Mutations in their encoding genes lead to DHDDS- and NUS1-CDG, respectively (Buczkowska et al., 2015). The polyprenol is then reduced by steroid 5 alpha reductases type 3 (SRD5A3) to dolichol. SRD5A3-CDG (previously known as CDG-Iq) (Canatgrel et al., 2010) (MIM:612379) is caused by deleterious mutations in the *SRD5A3* gene and in patients with SRD5A3-CDG, and an increase of polyprenols has been shown (Gründahl et al., 2012; Online Mendelian inherita, 2021). Dolichol is further phosphorylated by dolichol kinase (DOLK), and mutations in the *DOLK* gene cause DOLK-CDG. The phosphorylated dolichol might also act as a carrier for monosaccharides such as mannose and glucose used as donors in N-glycosylation as well as O-mannosylation, C-mannosylation, and GPI anchor synthesis.

SRD5A3-CDG is an ultrarare CDG subtype caused by autosomal recessive inheritance and has been reported so far in 38 patients. Key diagnostic features of SRD5A3-CDG are ophthalmological abnormalities such as retinitis pigmentosa/retinal dystrophy and optic nerve hypoplasia presenting at early onset. SRD5A3-CDG is also characterized by variable neurological symptoms including intellectual disability, cerebellar abnormalities, and hypotonia.

We report on the in-depth phenotyping of eleven SRD5A3-CDG patients of five sets of sibships that are followed up at our center. We compare their clinical and molecular findings to those cases reported in the literature.

## Materials and Methods

In total, 11 SRD5A3-CDG patients of five sets of sibships were included in the study. Data on 9 of 11 patients are yet unpublished. Siblings from family 5 were reported by Al-Gazali et al. (2008). Patients’ results on biochemical and genetic investigations and in-depth phenotyping were collected by retrospective review of the patients’ medical records and an additional questionnaire. Informed consent was taken from the legal representatives of previously unpublished cases. The clinical characteristics of these eight females and three male patients, with current ages ranging from 5 to 23 years, are summarized in Table 1. More detailed patient descriptions can be found in Supplementary Information S1.

**TABLE 1:** Clinical characteristics of patients with SRD5A3-CDG in our cohort of 11 patients.

Phenotypic presentation of 11 patients with SRD5A3-CDG											
Patient ID/Age	1-1/20 y	1-2/14.9 y	2-1/23 y	2-2/19 y	3-1/16 y	3-2/8.5 y	4-1/15 y	4-2/12 y	4-3/5 y	5-1/20 y*	5-2/17 y*
Gender	F	F	F	F	F	M	F	F	M	F	M
Ethnicity	Pakistani	Pakistani	Indian	Indian	Kurdish	Kurdish	Pakistani	Pakistani	Pakistani	Baluchi	Baluchi
Consanguinity	+	+	+	+	+	+	+	+	+	+	+
Age at onset (months)	9 m	Birth	18 m	Birth	2 m	6 m	6 weeks	3 weeks	6 weeks	Birth	Birth
Age at diagnosis (years)	15 y	2 y	16 y	12 y	11 y	4 y	13 y	8 y	4 y	∼5 y	∼1 y
Genetics	*SRD5A3 c.943C > T p.(Pro315Ser)* Homozygous	*SRD5A3 c.943C > T p.(Pro315Ser)* Homozygous	*SRD5A3 c.57G > A p.(Trp19Ter)* homozygous	*SRD5A3 c.57G > A p.(Trp19Ter)* homozygous	*SRD5A3 c.57G > A p.(Trp19Ter)* homozygous	*SRD5A3 c.57G > A p.(Trp19Ter)* homozygous	*SRD5A3 c.57G > A p.(Trp19Ter)* homozygous	*SRD5A3 c.57G > A p.(Trp19Ter)* homozygous	*SRD5A3 c.57G > A p.(Trp19Ter)* homozygous	*SRD5A3 p.Gln96delinsX* homozygous deletion	*SRD5A3 p.Gln96delinsX* homozygous deletion
Dysmorphic features	No	No	No	No	No	No	No	No	No	Yes	Yes
Ophthalmic findings	RD Squint	RD	RD, Nystagmus	RD, Nystagmus	RD Nystagmus Astigmatism	RD Nystagmus Astigmatism	RD Nystagmus	RD Nystagmus	RD Nystagmus Optic nerve hypoplasia	Squint Bilateral optic nerve colobomas	Bilateral iris and chorioretinal colobomas
Neuro-development	GDD, mild LD	GDD, severe LD, ASD	GDD, LD	GDD, LD	GDD, severe LD, ASD	GDD, severe LD, ASD	GDD, moderate LD	GDD, Mild LD	GDD, severe LD	GDD, LD	GDD, LD
Hypotonia	+	+	+	+	+	+	+	−	+	+	+
Seizures	−	−	−	−	−	−	−	−	−	−	−
Dystonia	−	−	−	−	−	+	−	−	+	−	−
Behavior	−	Anxiety	−	Anxiety	Anxiety	−	Mood swings	Anxiety	−	−	−
Ataxia	−	+	+	−	+	+	+	−	−	−	−
Cutaneous	Psoriasis	Ichthyosis, collodion baby	−	Mild hyperkeratosis	−	−	Eczema	Dry skin	−	Ichthyosis Hypertrichosis	Ichthyosis Hypertrichosis
Cardiac	Palpitations	Palpitations	−	−	AR, Prolonged QT	AR	−	−	−	TGA	sASD
Gastrointestinal	IBS	IBS, cyclical vomiting	−	Unexplained weight loss	−	Constipation, dairy allergy	−	−	Poor feeding, dysphagia, Gastrostomy	−	−
Renal	−	−	−	−	−	−	−	−	−	Right duplex kidney	−
Infection	−	−	−	−	−	Recurrent URTI and LRTI, on prophylactic azithromycin	−	−	Recurrent LRTI Prophylactic azithromycin	−	−
Endocrine	Primary Ovarian Failure	−	−	−	Irregular menstruation	−	−	−	−	Small AP, SOD	Small AP
Spinal scoliosis	−	−	+	+	+	+	+	−	+	−	−
Joint laxity	−	−	−	−	+	+	−	−	+	−	−
Dental	−	−	−	Crowding, difficult hygiene	−	−	Dental extractions	−	Premature loss of milk teeth	−	−
Investigations												
Transferrin isoelectric focusing	Abnormal type 1 pattern	Abnormal type 1 pattern	nk	Nk	Normal	Abnormal type 1 pattern	Abnormal type 1 pattern	Abnormal type 1 pattern	Abnormal type 1 pattern	Abnormal type 1 pattern	Abnormal type 1 pattern	
Clotting	Normal	Normal	nk	nk	Normal	Prol. APTT	nk	nk	nk	nk	nk	
TFT	Normal	Normal	nk	nk	Nk	Normal	nk	nk	Normal	Norm	Normal	
Testosterone	3.8 (H)	Nk	nk	nk	Nk	nk	nk	nk	nk	nk	nk	
FSH/LH	4.3/15.3	3.4/15.0	nk	nk	Normal	nk	nk	nk	nk	nk	nk	
GH	Nk	Normal	nk	nk	Nk	Normal	nk	nk	nk	Norm	nk	
Liver test	Normal	Normal	nk	Normal	Normal	Elevated	Normal	nk	Elevated	Norm	Elevated	
MRI brain	Findings suggestive of demyelination	Cavum septum pellucidum et vergae, small basal ganglia, mild cerebellar hypoplasia and dysplasia, thick corpus callosum, punctate white matter lesions	Not done	Not done	Normal apart from non-specific subcortical white matter foci in the frontal lobe	Malrotation of hippocampus, retro-cerebellar cyst, thin cervical cord, immature myelin, basal ganglia small (LN)	Not done	Not done	Small cerebral white matter volume, small cerebellum and pons, delayed myelination, small basal ganglia (LN), small optic nerves, malrotation of hippocampus	Small AP, left microphthalmia and atrophic visual pathways	Cerebellar vermis hypoplasia, b/l severe frontal microgyria, delayed myelination	

(+), present; (−), not present; (*), patients lost to follow up; RD, retinal dystrophy; GDD, global developmental delay; LD, learning difficulties; ASD, Autism spectrum disorder; AR, abnormal repolarisation; TGA, transposition of the great arteries; sASD, secundum atrial septal defect IBS, irritable bowel syndrome; URTI, upper respiratory tract infection; LRTI, lower respiratory tract infection; AP, anterior pituitary; SOD, septo-optic dysplasia; APTT, activate partial thromboplastin time; TFT, thyroid function; LN, lentiform nucleus; nk, not known.

## Results

### Demographics

A total of 11 patients with SRD5A3-CDG are included in our cohort, with a female to male ratio of 8:3. The current ages ranged from 5 to 23 years.

### Clinical Features

**FIGURE 1:**
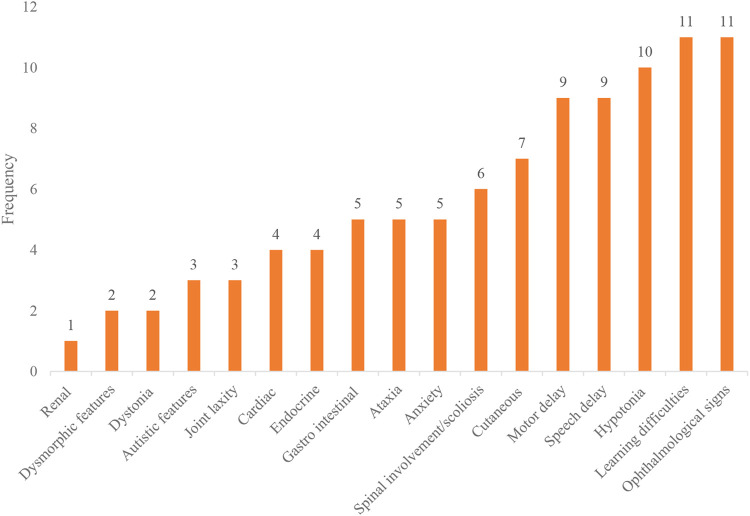
Frequency of symptoms affecting our SRD5A3-CDG cohort. This bar chart demonstrates the number of patients affected by each clinical feature/organ involvement. Learning difficulties (11/11), ophthalmological signs (11/11), hypotonia (10/11), speech delay (9/11), motor delay (9/11), cutaneous (7/11), scoliosis (6/11), anxiety (5/11), ataxia (5/11), gastrointestinal (5/11), cardiac (4/11), endocrine (4/11), autistic features (3/11), joint laxity (3/11), dystonia (2/11), dysmorphism (2/11), and renal (1/11).

**FIGURE 2:**
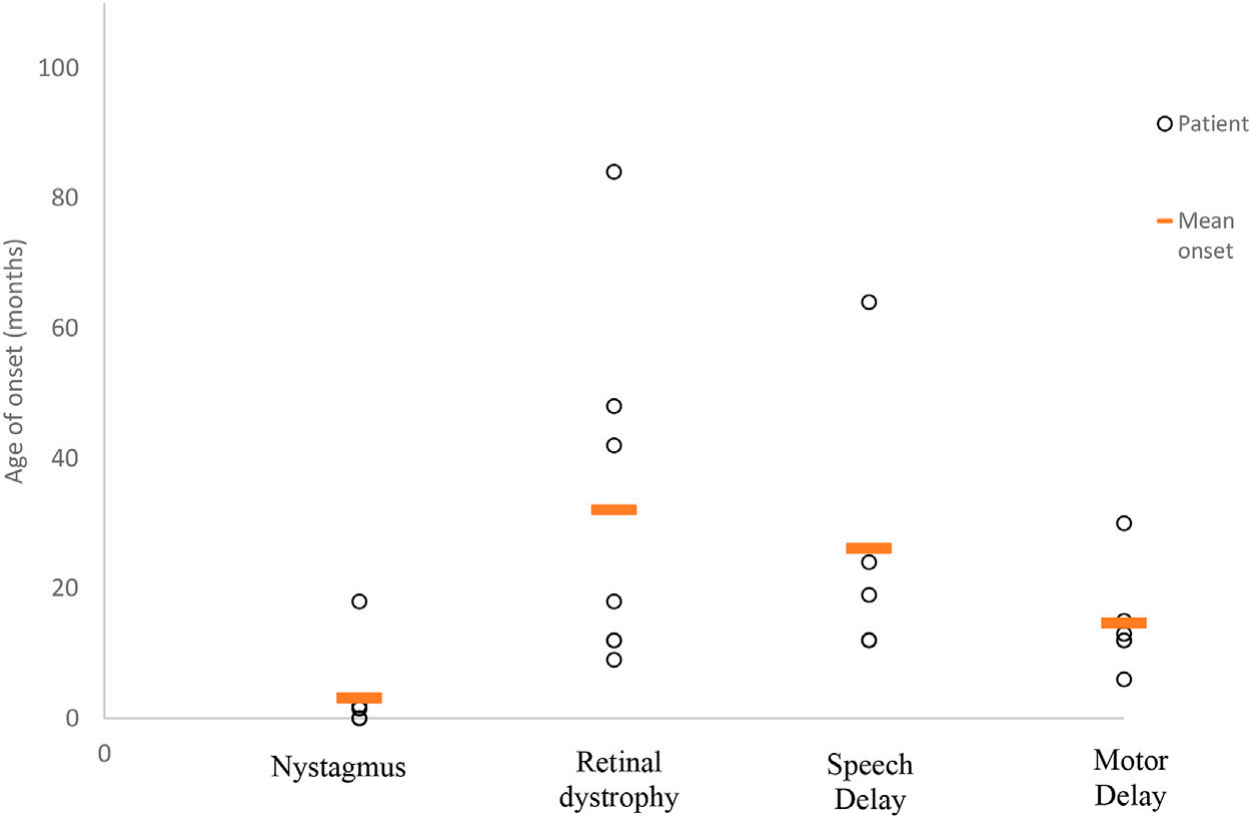
Average age at symptom onset. This scatter graph illustrates the timing of symptom onset ascertained from patient records as documented. Timing of symptom onset was not identifiable for all clinical features. Therefore, those included are as follows (number of patients/mean age of onset in months): nystagmus (6 of 7/3 months), retinal dystrophy (7 of 9/32 months), speech delay (5 of 9/26 months), and motor delay (6 of 9/15 months).

All 11 patients in our cohort came to the attention of clinicians due to parental concerns regarding developmental delay and visual abnormalities noted from as early as birth. Despite the early presentation of symptoms (average 3.5 months of age), the mean time to obtaining a diagnosis was 8 years (range 1–14.5 years) from symptom onset. The frequency of symptoms experienced by our cohort has been summarized in Figure 1, while in Figure 2, we illustrate the timing of onset of certain clinical features, which were identifiable from clinical documentation. The earliest clinical feature identified was nystagmus (average onset 3 months, range 0–18 months). This was followed by motor delay (average onset 15 months, range 6–30 months). Speech delay was a documented feature in nine patients with an onset of 26 months (range 12–64 months). Eye signs then progressed further to retinal dystrophy at an average onset of 32 months (range 9–84 months).

**FIGURE 3:**
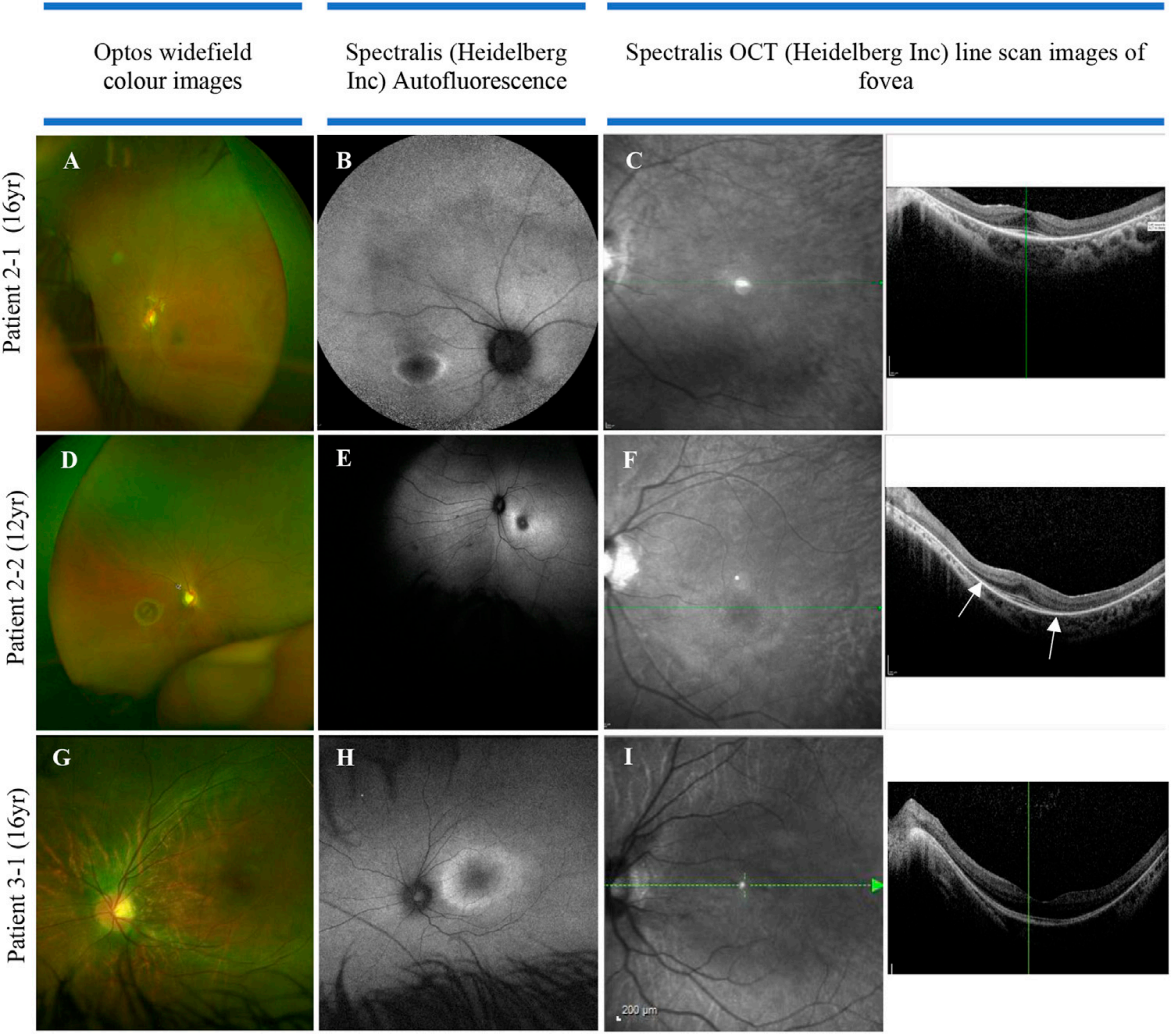
Color fundus, autofluorescence, infrared, and OCT images in three individuals of two families with SRD5A3-CDG. **(A)** Optos image of eye with nystagmus (leading to some distortion of the image), demonstrating mild vascular attenuation, myopic oval disc morphology, and subtle macula reflex abnormalities. The peripheral retina does not reveal any pigmentary abnormalities; **(B)** delineating “watershed” zone between relatively preserved central retina, and dystrophic periphery; **(C)** loss of ellipsoid outside the perifoveal region in both eyes; **(D)** myopic discs, retinal nerve fiber layer reflexes constrained to macula, subtle vascular attenuation, and no significant retinal pigmentation migration suggestive of retinal dystrophy. **(E)** Abnormal macula with hyper-autofluorescent ring around fovea; **(F)** loss of ellipsoid—the outer retinal layers beyond the fovea (as highlighted by arrows). **(G)** Color image of left eye; **(H)** hyper-autofluorescent ring illustrating watershed area between preserved central macula function and dystrophic retinal periphery; **(I)** loss of ellipsoid layer outside the perifoveal retina.

The multi-systemic, heterogeneous nature of SRD5A3-CDG is evident from the clinical features observed. Key diagnostic features are ophthalmic abnormalities experienced in all (11/11) individuals of our cohort, including nystagmus, retinal dystrophy, optic nerve hypoplasia, and colobomas. Non-specific visual symptoms such as nystagmus are present from very early onset (e.g., from birth), followed by early-onset retinal dystrophy. Figure 3 illustrates the ophthalmological findings in two of our families.

Both siblings in family 2 were born with signs of infantile-onset retinal dystrophy: they had a pendular nystagmus, photophobia, and reduced visual behaviors. Electrodiagnostic testing was difficult and limited, secondary to their developmental delay, but revealed attenuated responses to both rod and cone stimulation with delayed 30 Hz flicker, suggesting widespread retinal dysfunction. Their central vision has remained largely stable over the years, but the presence of a hyper-autofluorescent ring and the loss of the ellipsoid layer seen on OCT, outside the perifoveal region, indicate a widespread and significant retinal dystrophy. In patient 3-1, the OCT scans show loss of the photoreceptors (ellipsoid) inside the macula that was detectable from the age of 11. She had a dystrophy identified on electrodiagnostics from the age of 3; however, similar to family 2, she had an early-onset retinal dystrophy. In summary, the data from the two families in Figure 3 suggest a reasonably coherent phenotype: all have an early onset rod–cone dystrophy on electrodiagnostics, with a hyperfluorescent ring visible on fundus autofluorescence in their late teenage years indicating advanced retinal dystrophy. The progression of the disease, despite being onset during infancy, suggests that patients retain the capacity for moderate central vision into their 20s.

**FIGURE 4:**
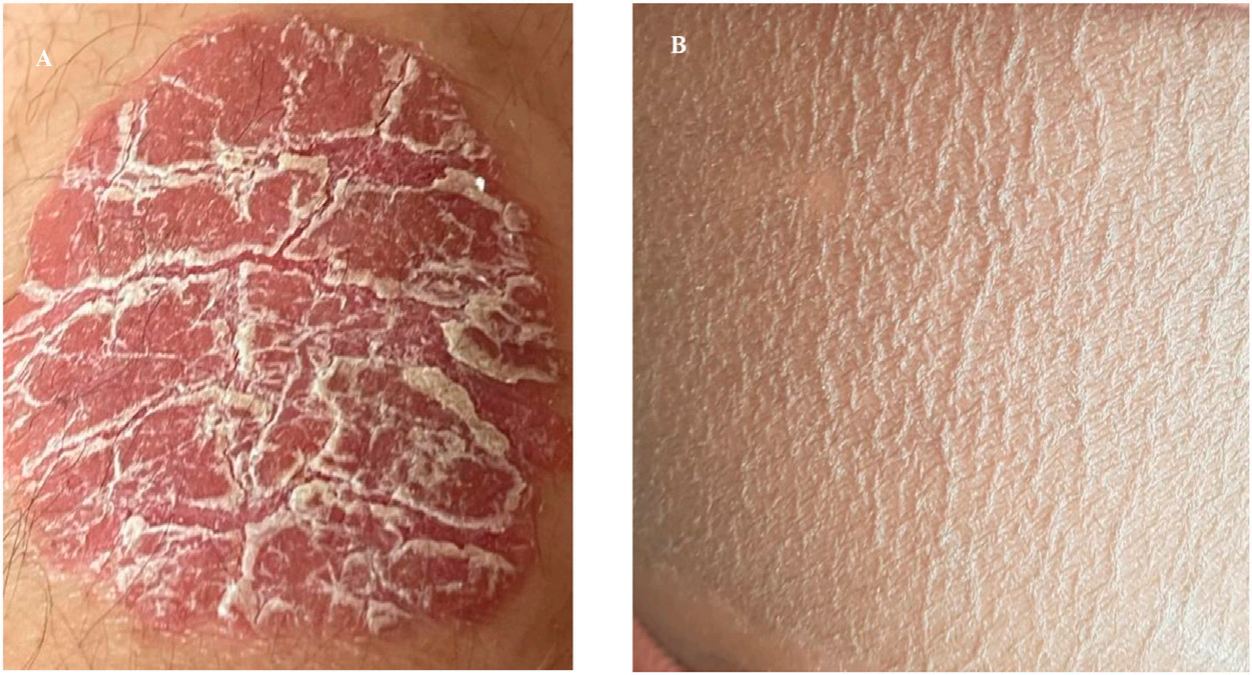
Examples of skin changes seen in SRD5A3-CDG. **(A)** Well demarcated erythematous and scaly plaque classical of psoriasis seen in patient 1–1. **(B)** Excessively dry scaly skin representative of ichthyosis seen in patient 1–2 who was born as a collodion baby.

SRD5A3-CDG is also characterized by variable neurological symptoms including hypotonia (10/11), ataxia (5/11), anxiety disorder (5/11), autism (3/11), and movement disorder (dystonia in 2/11). Patient 3-2 has also developed sensorimotor neuropathy. Global developmental delay and subsequent learning disabilities are experienced by all of our patients. Cutaneous and musculoskeletal features such as ichthyosis/psoriasis (4/11, Figure 4), scoliosis (6/11), and joint laxity (3/11) have been observed.

We have also seen that around half of our patients have gastrointestinal symptoms (5/11). Cardiac abnormalities have been described in 6/11 patients varying from structural abnormalities to conduction abnormalities and palpitations reported by patients. Interestingly dysmorphic features were only present in 2/11 patients. In two female patients from different families, we observed primary ovarian failure and menstruation irregularities. Pituitary abnormalities were seen in 2/11 patients. Comparing the clinical symptoms of siblings in our cohort of five families, with either two or three affected individuals, there is clear intra-familiar variability of signs and symptoms.

### Genetics

All our patients were diagnosed by non-targeted whole-exome sequencing and/or *via* the 100,000 genome project, a UK government project funded by the NHS. SRD5A3-CDG is an autosomal recessive disorder, though interestingly, in our cohort, all our families have affected siblings, where four out of five sibships were duos, and one family presented with three affected children. The commonest SRD5A3-CDG mutation reported so far is *c.57G > A p.(Trp19Ter) p.W19X*, also found in seven patients in our cohort (Morava et al., 2010; Gründahl et al., 2012; Kara et al., 2014; Tuysuz et al., 2016; Taylor et al., 2017; Gupta et al., 2018). These families are of Kurdish, Indian, or Pakistani background. Family 1, also of Pakistani origin, was found to be homozygous for the *c.943C > T p.(Pro315Ser)* variant. Interestingly this mutation has not yet been reported. Family 5, of Baluchi origin, were found to carry the *p.Gln96delinsX* homozygous deletion, which resulted in a frameshift and premature termination at amino acid 96 of 318 of the SRD5A3 protein.

### Laboratory

Isoelectric focusing of transferrin (IEF), the commonly used screening test for N-glycosylation disorders, was performed in nine patients and was found to be abnormal in 8. The abnormal IEF results showed a type I pattern, characterized by an increase of di- and asialotransferrin, pointing to an early assembly defect in the dolichol-linked glycosylation. Additional biochemical parameters, often found to be abnormal in CDG patients, were variably performed in at least 9/11 patients. These include thyroid function tests, liver enzymes, clotting, and sex hormones. We only observed one patient with a prolonged APTT that later normalized. Three patients had elevated liver enzymes but all patients investigated had normal thyroid function. Premature ovarian failure and irregularity of menstruation were reported for patients 1-1 and 3-1, respectively.

### Neuroimaging

**FIGURE 5:**
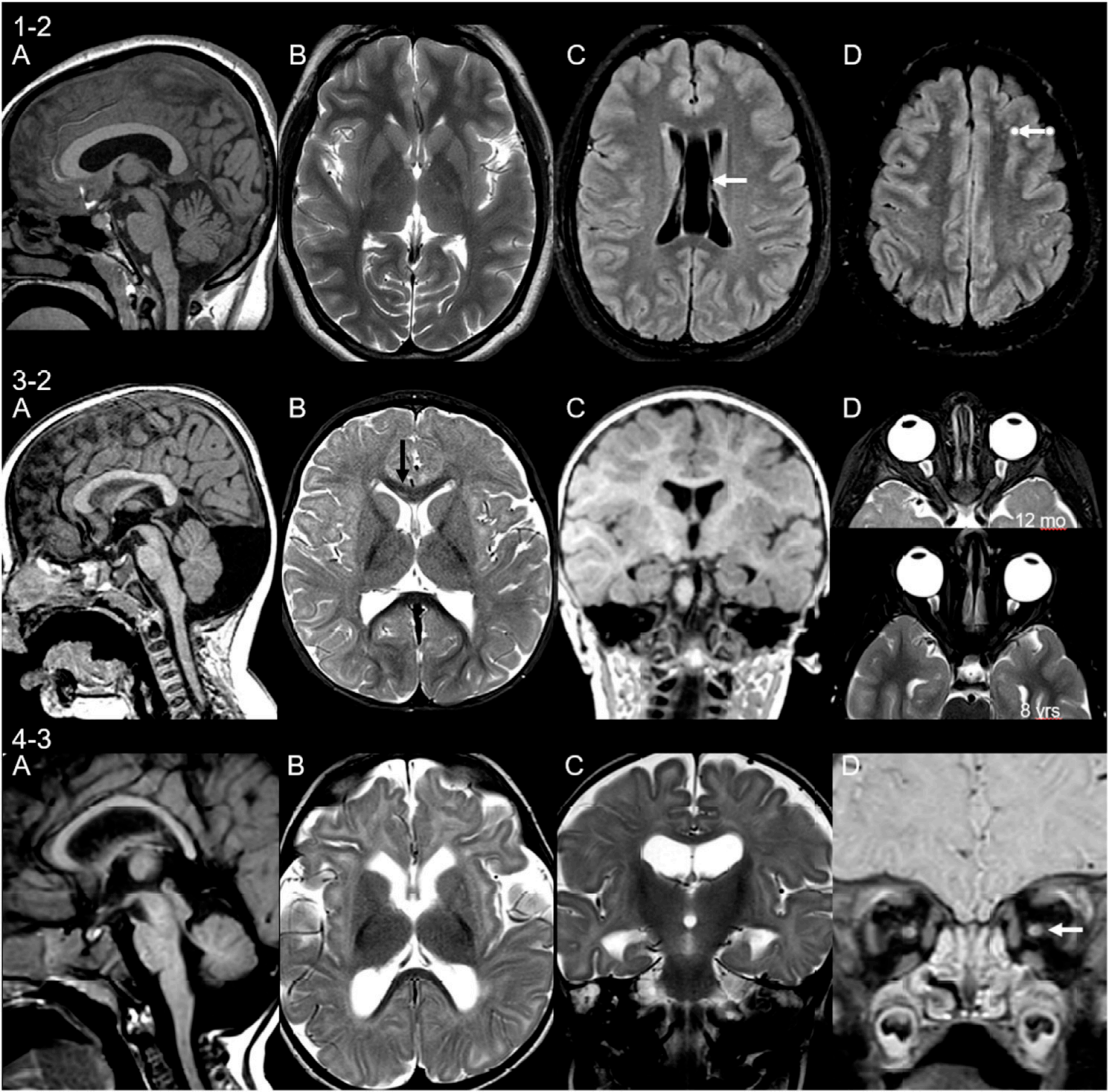
Neuroimaging findings in three unrelated patients. **MRI of patient 1-2** at age 15 years shows **(A)** thick corpus callosum and mild cerebellar hypoplasia, **(B)** small basal ganglia, **(C)** cavum septum pellucidum et vergae (arrow), and **(D)** non-specific punctate white matter lesions on fluid-attenuated inversion recovery (FLAIR) sequences (arrow). **Patient 3-2** scanned at 12 months of age shows **(A)** retrocerebellar cyst and thin cervical cord, **(B)** signal change of the genu of corpus callosum (immature myelin, black arrow) and small lentiform nucleus, **(C)** malrotation of hippocampi, and **(D)** asymmetry of the globes, more prominent on follow-up (lower image). **Patient 4-3** at 7 months of age shows **(A)** small cerebellum and pons and drooping splenium, **(B)** microcephaly with volume loss of the white matter, delayed myelination, and volume loss of the lentiform nucleus, **(C)** malrotation of hippocampi, and **(D)** volume loss of the optic nerves.

Neuroimaging findings were variable in our cohort with intra-familiar variability. MRI brain was performed in seven patients (four families). Detailed descriptions are found in Table 1 with MRI brain images in Figure 5. Findings included cerebellar and cerebellar vermis hypoplasia, severe frontal microgyria, delayed myelination, small cerebral white matter volume loss, drooping splenium and small pons, small basal ganglia (lentiform nucleus), and mal-rotated hippocampus. The latter two features have not previously been described in association with glycosylation disorders. Other findings include evidence of demyelination in patient 1-1, who has a second diagnosis of multiple sclerosis*.*

## Discussion

SRD5A3-CDG is an ultrarare CDG diagnosis with 38 cases reported so far (Jaeken et al., 2020). The clinical spectrum of SRD5A3-CDG is still evolving, and the diagnosis of patients is often only established by untargeted extended mutational screening. Delineating these features remains a challenge due to the small number of patients and limited individual case data available.

We present here the in-depth phenotyping of another nine unpublished cases of five sets of siblings with SRD5A3-CDG from unrelated families of different ethnic backgrounds. Transferrin isoelectric focusing (IEF), as a screening test for N-glycosylation defects, will usually show a type 1 pattern in SRD5A3-CDG. However, affected patients with a normal pattern have also been reported, as seen in patient 3-1. All our patients were diagnosed by non-targeted whole-exome sequencing and/or *via* the 100,000 genome project. Interestingly, despite early symptom onset, there is an average delay of almost 8 years between the first symptom and diagnosis. There is an apparent phenotypic variability in the clinical features among patients with SRD5A3-CDG. This variability interestingly extends to affected siblings within the same families, carrying the same genetic mutation as we demonstrate in our patient cohort. As each set of siblings comes from a background of consanguineous parents, there is an increased probability of additional inherited genetic factors contributing to the patients’ signs and symptoms. However, no other gene variants of particular concern were reported in our patients.

The ophthalmological findings in SRD5A3-CDG are already well delineated in the literature. Each of our patients is affected by eye changes which include nystagmus, retinal dystrophy, optic nerve hypoplasia, squint, and colobomas. In our cohort, nystagmus was the most common presenting feature early in infancy, and therefore an important though unspecific diagnostic sign. Seven of 11 patients were diagnosed with early-onset retinal dystrophy with onset as early as 9 months of age. This is comparable to a previous case series which reported early-onset (≤3 years) retinal dystrophy in SRD5A3-CDG patients carrying a common mutation (*c.57G > A p.(Trp19Ter)* homozygous) (Taylor et al., 2017). This is the same mutation carried by the seven of 11 patients in our cohort diagnosed with early-onset retinal dystrophy. Using our cohort, and those described in the literature, we can suggest that nystagmus followed by the development of early retinal dystrophy and visual impairment forms part of the natural disease history in SRD5A3-CDG patients. It has been hypothesized that hypo-glycosylation of rhodopsin expressed in rod photoreceptor cells could be responsible for the early onset changes on the retina. Rhodopsin has two N-glycosylation sides, and mutations in the *SRD5A3* gene leading to abnormal glycosylation of rhodopsin might affect its normal incorporation and function in the rod outer segment. This could then subsequently lead to defective phototransduction, loss of vision, and development of retinal dystrophy (Taylor et al., 2017). The other patients harboring different mutations also had profound retinal or structural changes of the eye. Siblings from family 1 presented with retinal dystrophy in early childhood, whereas patients in family 5 were found to have colobomas. Colobomas have been described in four other families each carrying different mutations in the *SRD5A3* gene (Canatgrel et al., 2010).

There is also some phenotypic overlap with other congenital disorders of glycosylation, resulting in an early disruption of the dolichol pathway: patients with *DHDDS-*CDG (MIM 613861) have been found to have isolated non-syndromic retinitis pigmentosa (Zelinger et al., 2011) or severe multisystem disease (Sabry et al., 2016). Suppression of DHDDS expression in zebrafish leads to the loss of photoreceptor’s outer segments and visual function, supporting the hypothesis that insufficient DHDDS function leads to retinal degeneration (Wen et al., 2014). In two siblings diagnosed with NUS1-CDG, alongside severe neurological impairment, epilepsy, and hearing deficit, these children had visual impairment with discrete bilateral macular lesions at the age of 4 years (Park et al., 2014). The spectrum of ocular involvement from early onset highlights the role and importance of the dolichol biosynthesis pathway in the normal development of the eyes.

Global developmental delay and learning difficulties are known features of SRD5A3-CDG, with each of our patients affected to some degree. There appears to be obvious variability among affected siblings; family numbers 1 and 4 are a good example of this with individuals on the severe spectrum of learning difficulties, in comparison to their siblings who are either requiring minimal support or managed to pursue higher education. Three patients with severe learning difficulties are also affected by autism spectrum disorder, two of whom are siblings. In addition, anxiety or mood alteration appears to be a feature in females, with over 60% (5/8) of our female cohort affected. Anxiety appears to be an evolving feature and may be at least partially secondary to visual impairment, and therefore affecting those with a lesser degree of intellectual disability and higher functionality. In contrast, anxiety may not be well recognized in those with a more severe intellectual disability and less independently functional, as all of our patients are affected by a degree of visual impairment.

Other neurological findings include hypotonia, and all except one of our cohort suffer from this to varying degrees, which is evident in their slow progression, or lack of progression of gross motor skills. It is hypothesized that mutations in the *SRD5A3* gene also disrupt O-mannosylation, which is essential for muscular tissue maintenance, which could be contributing to the muscular symptoms seen in our patient cohort (Endo, 2019). Only one patient in our cohort (4-1) has reported normal tone with normal mobility and posture despite her siblings being affected by marked hypotonia. Six of our patients with hypotonia have developed or have signs of early emerging scoliosis. The degree of scoliosis and its early onset in childhood may not be fully attributable to hypotonia, which is only mild in some of our cases. Two of our male patients described also suffer from dystonia requiring medical management. Dystonia might, therefore, be an additional neurological feature that needs to be evaluated in SRD5A3-CDG patients. Dystonia is rarely described in other CDG subtypes. Nevertheless, a single case report from Prietsch et al. (2002) describes a female patient who developed dystonic hand movements, and dystonia has been described in cases of PMM2-CDG (Prietsch et al., 2002; Mostile et al., 2019). Patient 3-2 has also developed a sensorimotor neuropathy, a feature not previously described in this ultrarare condition, and possibly also needs to be recognized as a new manifestation of this disorder.

The variable frequency and diversity of brain developmental abnormalities is a known feature in CDGs, and this is demonstrated in our cohort. Proper protein glycosylation is essential for normal brain development and function, thereby explaining the variety of features seen in CDG patients. Malformations described include cortical malformations, midline brain structure and volume anomalies, myelination disorders, and venous sinus thrombosis (Paprocka et al., 2021). Classically, type 1 CDG patients demonstrate patterns of cerebellar, pontocerebellar, and cerebellar vermis hypoplasia and atrophy. Cantagrel et al. (2010) summarized MRI findings of five families with the loss of function mutations in the *SRD5A3* gene that were found to have signs of cerebellar atrophy or vermis malformations (Canatgrel et al., 2010). This may provide an explanation for the early onset nystagmus as well as ataxia seen in SRD5A3-CDG patients; we see a high prevalence of ataxia in our cohort (5/11) with intra-familiar variability. Medina-Cano et al. (2018) observed motor coordination defects and abnormal granule cell development in a cerebellum-specific knockout mouse for *SRD5A3*. Their proteomic studies confirmed that *SRD5A3* loss affects a specific subset of glycoproteins, namely, those highly glycosylated. They observed, for example, impaired IgSF-CAM–mediated neurite growth and axon guidance in the cerebellum of their *SRD5A3* mutant mice (Medina-Cano et al., 2018). Interestingly, in addition to the central nervous system, highly glycosylated IgSF-CAM members also play critical roles in other systems such as the developing eye (Morava et al., 2009), which might explain both the cerebellar and ocular signs and symptoms in patients with SRD5A3-CDG. The finding of severe frontal microgyria in patient 5-2 is intriguing. Neuronal migration defects are common features of a subgroup of O-glycosylation defects, namely, O-mannosylation defects leading to dystroglycanopathies. As O-mannosylation might also be hampered in SRD5A3-CDG, this might contribute to the misfolding of the cortex (Schiller et al., 2020). The findings of the mal-rotated hippocampus and small brainstem are features not previously described in SRD5A3-CDG or other glycosylation defects. The cause of a mal-rotated hippocampus itself is unexplained although thought to be a result of the failure of the normal infolding process during development *in utero*. The mechanism, if this failure is unclear, could be a result of acquired or genetic factors (Fu et al., 2021). White matter abnormalities are not a typical characteristic in CDGs although they have been described in only a few patients (Paprocka et al., 2021). Similarly in our cohort, white matter volume loss was only described in a single patient.

In total, 5 of 11 patients in our cohort displayed skin changes, and among those affected, symptoms varied from generalized dryness and eczematous changes to psoriasis and ichthyosiform changes. At the severe end of the spectrum, a single patient presented at birth with ichthyosis with troublesome symptoms in infancy easing with older age (1–2). Interestingly, his sibling, affected with the same genetic mutation, has a milder cutaneous involvement with the development of psoriasis at a later age. Variable skin involvement has been previously reported, and the most common findings are those of ichthyosiform changes, hyperpigmentation, and palmoplantar keratoderma (Wheeler et al., 2016). The very first steps in the dolichol synthesis pathway share those of the sterol pathway up to the formation of farnesyl-PP. It has been suggested that the accumulation of toxic sterol precursors could contribute to the cutaneous phenotype of SRD5A3-CDG, and could also explain ichthyosis being observed in DOLK-CDG (Rymen and Jaeken, 2014).

Gastroenterological symptoms have been reported in 5/11 patients in our cohort. Siblings in family 1 have symptoms of presumed irritable bowel syndrome. The youngest sibling in family 4, with a more severe phenotype, was noted to have difficulties with feeding from 3 weeks of age, and he had episodes of recurrent vomiting and was gastrostomy fed from approximately 1 year of age. He also suffers from severe hypotonia and remains immobile. The *SRD5A3* gene is expressed in the duodenum so disruption of its function might impact gut mucosa maintenance, intestinal motility, and absorption contributing to this phenotype (Morava et al., 2010). As these symptoms are rather non-specific, gastrointestinal symptoms could be an under-reported feature.

Cardiac phenomena have been described only sporadically in cases of SRD5A3-CDG. Though in our cohort, we have 6/11 patients with cardiac involvement varying from clinical symptoms and conduction abnormalities to structural abnormalities. Siblings from family 1 are symptomatic of palpitations although cardiac investigations have been normal so far. Patient 1-2 also suffers from anxiety of which palpitations may be a manifestation. There is a history of abnormal repolarisation in family 3, and this includes unaffected family members. Siblings in family 5 have a history of structural heart defects as described. As the *SRD5A3* gene is expressed in cardiac tissue, and although its exact role is yet to be understood, its impact on dolichol synthesis and N- and O-glycosylation within cardiac tissue could explain these emerging phenotypes (Morava et al., 2010).

Endocrine dysfunction, particularly hypergonadotropic hypogonadism in CDG disorders, has been described in the literature, and the patient’s pubertal development should be monitored (Miller and Freeze, 2003). Interestingly, patient 1-1 has primary ovarian failure, and 3-1 has irregular menstrual periods, which need further investigation. Siblings in family 5 have evidence of small anterior pituitary glands with patient 5-1 suffering from a variation of septo-optic dysplasia. Growth abnormalities have not been described in our cohort.

It is questionable if the uncommonly described feature of recurrent infections is linked to the diagnosis of SRD5A3-CDG. Two of our patients from unrelated families have a history of recurrent upper and lower respiratory tract infections requiring prophylactic azithromycin. Patient 3-2 has been extensively investigated for underlying immune deficiencies, of which all results are normal. There was symptomatic improvement in patient 4-3 after gastrostomy insertion, suggesting recurrent aspiration from unsafe swallow was the likely cause for these recurrent lower respiratory tract infections.

Isoelectric focusing (IEF) of transferrin is commonly used to screen for glycosylation disorders. In our patient cohort, eight out of nine patients that had IEF showed a type I pattern, whereas in one family the older sibling had a normal result. This phenomenon has been seen in other patients with SRD5A3-CDG, so normal glycosylation screening does not rule out SRD5A3-CDG. Variable and age-dependent changes in glycosylation patterns have also been reported in other CDG subtypes (Mohamed et al., 2011).

In summary, patients with SRD5A3-CDG present with signs and symptoms of early ophthalmological abnormalities, developmental delay and intellectual disability, neurological symptoms, cutaneous changes, and spinal involvement, and fewer described cardiac and endocrine manifestations. Furthermore to the range of symptoms already prescribed, we report on emerging additional manifestations such as dystonia, anxiety disorder, and gastrointestinal symptoms. The genotypic and phenotypic spectrum of *SRD5A3*-CDG is evolving, and we also note intra-familiar variability. Genotype-phenotype analyses are hampered due to the limited number of patients being diagnosed so far. In addition, some of those diagnosed carry compound heterozygous mutations, making it even more difficult to predict an outcome. The presence of residual enzyme activity in some patients could provide an explanation for the different severity of patients’ presentation, and it has also been suggested that there might be an alternative biosynthetic pathway for dolichol synthesis in patients (Canatgrel et al., 2010). Mutant mouse model studies have shown the activation of the mevalonate pathway suggesting a positive feedback mechanism to overcome the lack of dolichol synthesis in some *SRD5A3* mutants by increasing substrate production (Canatgrel et al., 2010). Overall, SRD5A3-CDG manifests on a wide range of organs, which is likely to reflect the fact that *SRD5A3* gene mutations not only affect N-glycosylation but as dolichol is also required for the synthesis of O-mannose linked glycans, C-mannosylation, and glycophospholipid anchor synthesis, and disruption in these pathways may also explain part of the phenotypic spectrums. Further studies on cell and animal models and delineation of disrupted glycosylation pathways in humans will provide better insight into the different pathology of signs and symptoms in SRD5A3-CDG patients.

## Conclusion

The detailed description of the phenotype of this large cohort of patients with SRD5A3-CDG highlights that the key clinical diagnostic features of SRD5A3-CDG are early onset of ophthalmological problems. However, SRD5A3-CDG is a multi-systemic disorder also characterized by variable neurological symptoms including intellectual disability, hypotonia, and ataxia. In addition, we demonstrate the presence of cutaneous lesions as well as spinal, cardiac, and endocrine involvement. In our study population, new emerging clinical features include dystonia, anxiety disorder, gastrointestinal symptoms, and MRI findings of small basal ganglia and mal-rotated hippocampus. Dysmorphic features have been key to SRD5A3-CDG cases described in the literature, in contrast to our nine new patients where dysmorphic faces are not described. Furthermore, we newly observe clear intra-familiar variability in our cohort. This, alongside the evolving clinical spectrum of SRD5A3-CDG, makes it even more difficult to predict long-term prognosis, and there is a need for long-time follow-up of these patients. SRD5A3-CDG and other CDG subtypes in the early dolichol pathway should be considered as a cause of early-onset retinal dystrophy, particularly if patients present with multisystem disease. This would aid early diagnosis, inform genetic counseling, and would eventually provide patients access to emerging therapies. Disease-specific registries will help to capture the disease-specific manifestations and are particularly essential in such an ultrarare CDG subtype. Further delineation of the phenotypic spectrum should help to aid earlier diagnosis and guide clinicians to target long-term follow-up of SRD5A3-CDG patients.

## Data Availability

The original contributions presented in the study are included in the article/Supplementary Materials; further inquiries can be directed to the corresponding authors.

## References

[B1] Al-Gazali L. Hertecant J. Algawi K. El Teraifi H. Dattani M. (2008). A New Autosomal Recessive Syndrome of Ocular Colobomas, Icthyosis, Brain Malformations and Endocrine Abnormalities in an Inbred Emirati Family. Am. J. Med. Genet. Part. A. 146A, 813–819. 10.1002/ajmg.a.32114 18271001

[B2] Buczkowska A. Swiezewska E. Lefeber D. J. (2015). Genetic Defects in Dolichol Metabolism. J. Inherit. Metab. Dis. 38 (1), 157–169. 10.1007/s10545-014-9760-1 25270028 PMC4281381

[B3] Canatgrel V. Lefeber D. J. Ng B. G. Guan Z. Silhavy J. L. Bielas S. L. (2010). SRD5A3 Is Required for Converting Polyprenol to Dolichol and Is Mutated in a Congenital Glycosylation Disorder. Cell 142 (2), 203–217. 10.1016/j.cell.2010.06.001 20637498 PMC2940322

[B4] Endo T. (2019). Mammalian O-Mannosyl Glycans: Biochemistry and Glycopathology. Proc. Jpn. Acad. Ser. B, Phys. Biol. Sci. 95 (1), 39–51. 10.2183/pjab.95.004 PMC6395781 30643095

[B5] Fu T. Y. Ho C. R. Lin C. H. Lu Y. T. Lin W. C. Tsai M. H. (2021). Hippocampal Malrotation: A Genetic Developmental Anomaly Related to Epilepsy? Brain Sci. 11 (4), 463. 10.3390/brainsci11040463 33916495 PMC8067421

[B6] Gründahl J. E. H. Guan Z. Rust S. Reunert J. Müller B. Du Chesne I. (2012). Life with Too Much Polyprenol: Polyprenol Reductase Deficiency. Cell Mol. Genet. Metab. 105 (4), 642–651. 10.1016/j.ymgme.2011.12.017 22304929 PMC3428379

[B7] Gupta N. Verma G. Kabra M. Bijarnia-Mahay S. Ganapathy A. (2018). Identification of a Case of SRD5A3-Congenital Disorder of Glycosylation (CDG1Q) by Exome Sequencing. Indian J. Med. Res. 147, 422–426. 10.4103/ijmr.IJMR_820_16 29998879 PMC6057243

[B8] Jaeken J. Lefeber D. J. Matthijs G. (2020). SRD5A3 Defective Congenital Disorder of Glycosylation: Clinical Utility Gene Card. Eur. J. Hum. Genet. 28 (9), 1297–1300. 10.1038/s41431-020-0647-3 32424323 PMC7609305

[B9] Kara B. Ö Ayhan. Gökçay G. Başboğaoğlu N. Tolun A. (2014). Adult Phenotype and Further Phenotypic Variability in SRD5A3-CDG. BMC Med. Genet. 15, 10. 10.1186/1471-2350-15-10 24433453 PMC3898029

[B10] Medina-Cano D. Ucuncu E. Nguyen L. S. Nicouleau M. Lipecka J. Bizot J.-C. (2018). High N-Glycan Multiplicity Is Critical for Neuronal Adhesion and Sensitizes the Developing Cerebellum to N-Glycosylation Defect. Elife 7, e38309. 10.7554/eLife.38309 30311906 PMC6185108

[B11] Miller B. S. Freeze H. H. (2003). New Disorders in Carbohydrate Metabolism: Congenital Disorders of Glycosylation and Their Impact on the Endocrine System. Rev. Endocr. Metab. Disord. 4 (1), 103–113. 10.1023/a:1021883605280 12618564

[B12] Mohamed M. Cantagrel V. Al-Gazali L. Wevers R. A. Lefeber D. J. Morava E. (2011). Normal Glycosylation Screening Does Not Rule Out SRD5A3-CDG. Eur. J. Hum. Genet. 19, 1019. 10.1038/ejhg.2010.260 21750573 PMC3190245

[B13] Morava E. Wevers R. A. Cantagrel V. Hoefsloot L. H. Al-Gazali L. Schoots J. (2010). A Novel Cerebello-Ocular Syndrome with Abnormal Glycosylation Due to Abnormalities in Dolichol Metabolism. Brain 133, 3210–3220. 10.1093/brain/awq261 20852264 PMC6276930

[B14] Morava E. Wosik H. N. Sykut-Cegielska J. Adamowics M. Guillard M. Wevers R. A. (2009). Ophthalmological Abnormalities in Children with Congenital Disorders of Glycosylation Type I. Br. J. Ophthalmol. 93, 350–354. 10.1136/bjo.2008.145359 19019927

[B15] Mostile G. Barone R. Nicoletti A. Rizzo R. Martinelli D. Sturiale L. (2019). Hyperkinetic Movement Disorders in Congenital Disorders of Glycosylation. Eur. J. Neurol. 26 (9), 1226–1234. 10.1111/ene.14007 31132195

[B16] Ondruskova N. Cechova A. Hansikova H. Honzik T. Jaeken J. (2021). Congenital Disorders of Glycosylation: Still “hot” in 2020. Biochim. Biophys. Acta Gen. Subj. 1865 (1), 129751. 10.1016/j.bbagen.2020.129751 32991969

[B17] Online Mendelian inheritance in Man (2021). Online Mendelian Inheritance in Man, OMIM. Baltimore, MD: Johns Hopkins University. MIM Number: {# 612379} Marla J. F. O'Neill : 10/29/2008 , Cassandra L. Kniffin - updated : 9/9/2010.

[B18] Paprocka J. Jezela-Stanek A. Tylki-Szymańska A. Grunewald S. (2021). Congenital Disorders of Glycosylation from a Neurological Perspective. Brain Sci. 11 (1), 88. 10.3390/brainsci11010088 33440761 PMC7827962

[B19] Park E. J. Grabińska K. A. Guan Z. Stránecký V. Hartmannová H. Hodaňová K. (2014). Mutation of Nogo-B Receptor, a Subunit of Cis-Prenyltransferase, Causes a Congenital Disorder of Glycosylation. Cell Metab. 20 (3), 448–457. 10.1016/j.cmet.2014.06.016 25066056 PMC4161961

[B20] Prietsch V. Peters V. Hackler R. Jakobi R. Assmann B. Fang J. (2002). A New Case of CDG-X with Stereotyped Dystonic Hand Movements and Optic Atrophy. J. Inherit. Metab. Dis. 25, 126–130. 10.1023/a:1015628810892 12118527

[B21] Rymen D. Jaeken J. (2014). Skin Manifestations in CDG. J. Inherit. Metab. Dis. 37 (5), 699–708. 10.1007/s10545-014-9678-7 24554337

[B22] Sabry S. Vuillaumier-Barrot S. Mintet E. Fasseu M. Valayannopoulos V. Héron D. (2016). A Case of Fatal Type I Congenital Disorders of Glycosylation (CDG I) Associated with Low Dehydrodolichol Diphosphate Synthase (DHDDS) Activity. Orphanet J. Rare Dis. 11 (1), 84. 10.1186/s13023-016-0468-1 27343064 PMC4919849

[B23] Schiller S. Rosewich H. Grünewald S. Gärtner J. (2020). Inborn Errors of Metabolism Leading to Neuronal Migration Defects. J. Inherit. Metab. Dis. 43 (1), 145–155. 10.1002/jimd.12194 31747049

[B24] Taylor R. L. Gavin A. Poulter J. A. Khan K. N. Morarji J. Hull S. (2017). Association of Steroid 5α-Reductase Type 3 Congenital Disorder of Glycosylation with Early-Onset Retinal Dystrophy. JAMA Ophthalmol. 135 (4), 339–347. 10.1001/jamaophthalmol.2017.0046 28253385

[B25] Tuysuz B. Pehlivan D. Özkök A. Jhangiani S. Yalcinkaya C. Zeybek Ç. A. (2016). Phenotypic Expansion of Congenital Disorder of Glycosylation Due to SRD5A3 Null Mutation. JIMD Rep. 26, 7–12. 10.1007/8904_2015_478 26219881 PMC4864711

[B26] Wen R. Dallman J. E. Li Y. Züchner S. L. Vance J. M. Peričak-Vance M. A. (2014). Knock-down DHDDS Expression Induces Photoreceptor Degeneration in Zebrafish. Adv. Exp. Med. Biol. 801, 543–550. 10.1007/978-1-4614-3209-8_69 24664742

[B27] Wheeler P. G. Ng B. G. Sanford L. Sutton V. R. Bartholomew D. W. Pastore M. T. (2016). SRD5A3-CDG: Expanding the Phenotype of a Congenital Disorder of Glycosylation with Emphasis on Adult Onset Features. Am. J. Med. Genet. A. 170 (12), 3165–3171. 10.1002/ajmg.a.37875 27480077 PMC5115938

[B28] Zelinger L. Banin E. Obolensky A. Mizrahi-Meissonnier L. Beryozkin A. Bandah-Rozenfeld D. (2011). A Missense Mutation in DHDDS, Encoding Dehydrodolichyl Diphosphate Synthase, Is Associated with Autosomal-Recessive Retinitis Pigmentosa in Ashkenazi Jews. Am. J. Hum. Genet. 88 (2), 207–215. 10.1016/j.ajhg.2011.01.002 21295282 PMC3035703

