# Supplementary material for: SRD5A3-CDG: Emerging Phenotypic Features of an Ultrarare CDG Subtype
Source: Front Genet. 2021 Dec 1;12:737094. doi: 10.3389/fgene.2021.737094 (PMC8671882; doi:10.3389/fgene.2021.737094)

Figure 2 - Average age at symptom onset

This scatter graph illustrates the timing of symptom onset ascertained from patient records where documented. Timing of symptom onset was not identifiable for all clinical features. Therefore, those included are as follows (number of patients/ mean age of onset in months): nystagmus (6 of 7/ 3 months), retinal dystrophy (7 of 9/ 32 months), speech delay (5 of 9/ 26 months), and motor delay (6 of 9/ 15 months).

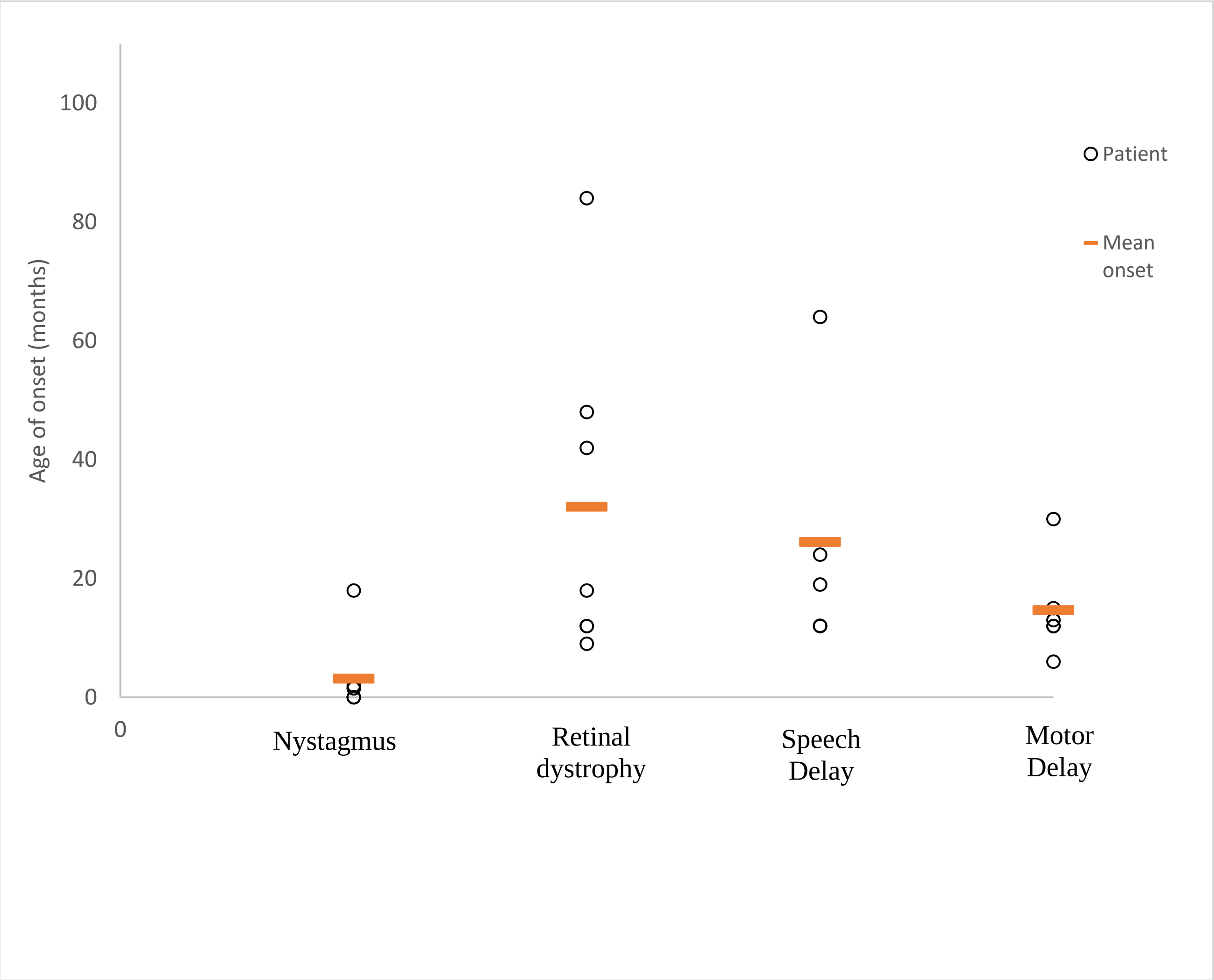

Supplement: Supplementary file 1 [file DataSheet2.pdf]
