# Supplementary material for: SRD5A3-CDG: Emerging Phenotypic Features of an Ultrarare CDG Subtype
Source: Front Genet. 2021 Dec 1;12:737094. doi: 10.3389/fgene.2021.737094 (PMC8671882; doi:10.3389/fgene.2021.737094)

Figure 4: Examples of skin changes seen in SRD5A3-CDG. (A) Well demarcated erythematous, scaly plaque classical of psoriasis seen in patient 1-1. (B) Excessively dry scaly skin representative of ichthyosis seen in patient 1-2 who was born as a collodion baby.

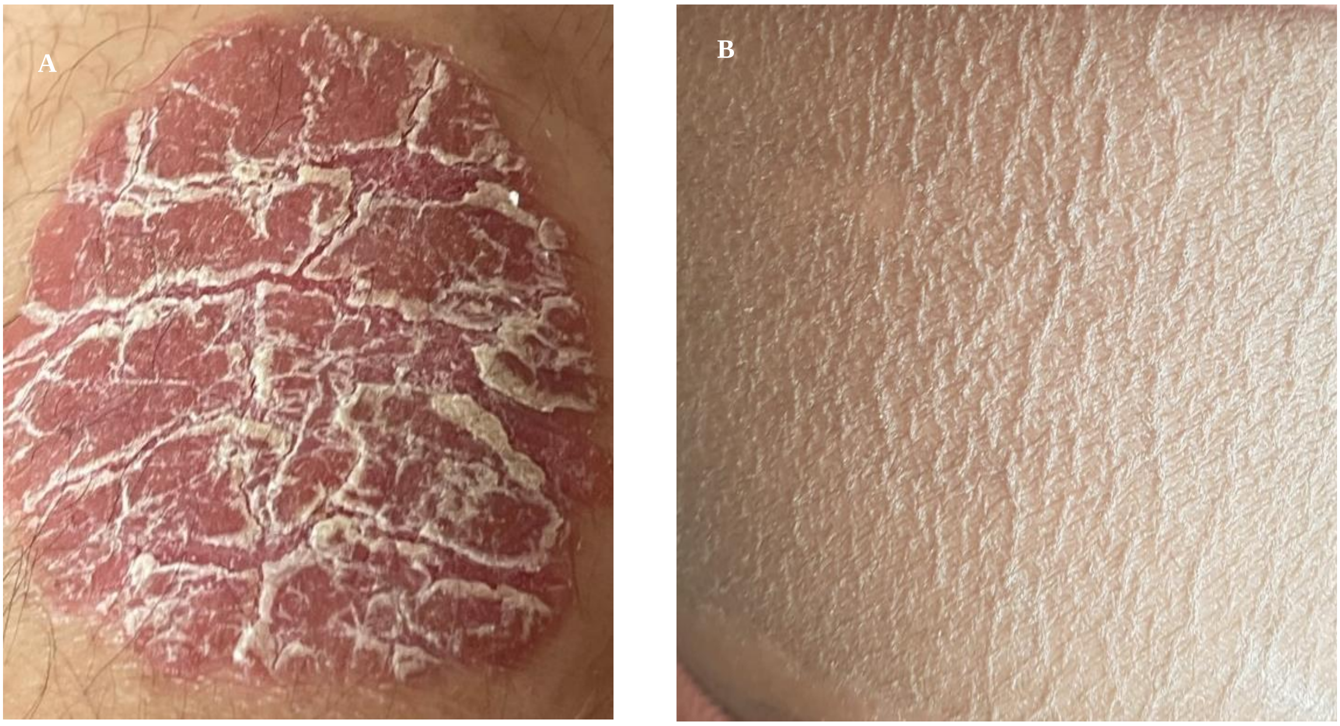

Supplement: Supplementary file 2 [file DataSheet4.pdf]
