# Supplementary material for: SRD5A3-CDG: Emerging Phenotypic Features of an Ultrarare CDG Subtype
Source: Front Genet. 2021 Dec 1;12:737094. doi: 10.3389/fgene.2021.737094 (PMC8671882; doi:10.3389/fgene.2021.737094)
Supplement: Supplementary file 3 [file DataSheet6.docx]

SUPPLEMENTARY INFORMATION

**Family 1**

Family 1 are Pakistani in origin. There is known consanguinity as well as a family history of ocular abnormalities (retinal dystrophy). **Patient 1-1** was born at term by spontaneous normal vaginal delivery. There were no problems during pregnancy or in the immediate post-natal period, although she was described as slightly floppy. At 9 months of age a squint was noted and following ophthalmology assessment retinal dystrophy was diagnosed. This has resulted in some degree of visual impairment. In terms of development, there was no evidence of speech delay however mild learning difficulties were evident, though not deterring her from mainstream education. She reached her motor milestones appropriately and walked at 1 year of age. Mild hypotonia with generalised reduced muscle strength appears to be an ongoing feature at the age of 20 years. Interestingly a diagnosis of multiple sclerosis was made following symptoms of headache and difficulty with handwriting at the age of 18 years. She remains fully independent with her self-care skills. There are some concerns about a possible evolving scoliosis. Symptoms of anxiety are experienced on a regular basis, and possibly associated with the occurrence of palpitations. Cardiac investigations are normal. Gastrointestinal symptoms have been described as she is experiencing increased frequency in defecation, thought to be irritable bowel syndrome. Cutaneous features include widespread psoriasis. Patient 1-1 has grown appropriately in height however by 14 years of age she was diagnosed with primary ovarian failure after initially reaching menarche aged 8 years.

**Patient 1-2** is the younger sibling of 1-1 and appears to have a more severe phenotype in comparison. She was born at 32 weeks’ gestation as a collodion baby, diagnosed with ichthyosis from birth, and has areas of hyperpigmentation. Her developmental milestones were delayed from early on. She was noted to be microcephalic and hypotonic. She walked from 5 years of age and had poor antigravity movements. She now has a broad-based, ataxic gait. Her MRI brain reveals cavum septum pellucidum et vergae, small basal ganglia, mild cerebellar hypoplasia and dysplasia, thick corpus callosum, and punctate white matter lesions. Currently, at the age of 14 years, she has significant learning difficulties, is able to speak short sentences only, with limited reading and writing skills. There are also behavioural concerns including lack of stranger awareness and other features consistent with autism spectrum disorder which has been diagnosed. Retinal dystrophy was diagnosed at 7 years of age, and impacts her vision and mobility. Like her sister she has symptoms of anxiety, with associated palpitations but normal cardiac investigations. She can eat and drink but suffers from cyclical episodes of vomiting and abdominal pain with increased frequency in defecation. Her growth is reported to be normal, and she has a regular menstrual cycle.

**Family 2:**

Patient 2-1 and 2-2 are sisters born to first- cousin consanguineous parents of Indian origin. There is no significant family history. **Patient 2-1** was born at term by spontaneous normal delivery. There were no antenatal or post-natal complications. Developmental concerns arose from 18 months of age where she was noted to have nystagmus and myopia and later diagnosed with retinal dystrophy. Between the ages of 2-3 years there was evidence of motor delay; she first walked at 3 years of age. Hypotonia and generalised weakness continue to manifest and a walking aid is required. Her gait is noted to be ataxic. Speech delay was mild and she is bilingually fluent. Learning difficulties were evident from early in childhood, however she remains independent with eating, and self-care skills. At the age of 23 years there are no other general health concerns and growth and pubertal development are reported to be normal.

**Patient 2-2** is the younger sibling of 2-1. She was born after an uneventful pregnancy and her clinical features followed similarly to her sister. Nystagmus was noted shortly after birth and later retinal dystrophy was diagnosed. She was delayed in her developmental milestones and there was generally slow progress in terms of gross motor, fine motor and speech development. She has learning difficulties as well as difficulty with short-term memory and significant anxiety with great impact on her ability to cope with basic skills, requiring mental health care involvement. Muscular tone is reportedly normal however there are some difficulties with balance and walking aids are required due to general weakness. There is some dry skin with a patch of hyperkeratosis on the lower limb. Gastrointestinal symptoms are non-specifically described as unexplained weight loss, but otherwise no other medical health concerns are reported. Growth and puberty are normal currently at 19 years of age.

**Family 3:**

Patient 3-1 and 3-2 are siblings born to healthy, consanguineous parents of Kurdish origin. **Patient 3-1** was born at term by caesarean section, in good condition weighing over 4.3kg. There were no antenatal or post-natal concerns. From 2 months of age nystagmus and astigmatism were diagnosed, and by the age of 3 years there was evidence of retinal dystrophy. She walked at 15 months of age however gross and fine motor development are delayed. Currently at 16 years of age there is difficulty walking due to hypotonia, ataxia, and laxity of ligaments with evidence of tiring on physical exertion. She is requiring a wheelchair for longer distances. Speech is delayed and she is semi- independent with self-care skills. There are significant learning difficulties with limited reading and writing skills, and background of autism spectrum disorder diagnosed earlier in childhood. Anxiety is significant and can cause restriction in dietary intake. Other systemic features include a history of prolonged QT with abnormal repolarisation evident on electrocardiograms; this is under cardiology follow up. Postural scoliosis and pronounced lordosis is also a feature. In her late teens there are persistent milk teeth with a history if difficult hygiene. She has reached menarche, however her menstrual cycle is irregular, warranting further investigation. MRI brain reveals nonspecific subcortical white matter foci in the frontal lobe.

**Patient 3-2** is the sibling of 3-1 and has a more severe phenotype in comparison. He was born at 39 weeks gestation by elective c section weighing 3.6kg. Post-natally he suffered from jaundice and significant weight loss secondary to feeding difficulties and cow’s milk protein intolerance. Developmental delay was evident from infancy, and like his sister, he has also been diagnosed with autism spectrum disorder. Speech remains delayed at 8.5 years of age as he uses echolalia and or vocalises incomprehensible sounds. He walked at the age of 2.5 years and remains ataxic and hypotonic, requiring wheelchair use for longer distances. There is also some joint laxity. Interestingly he has also developed a mild dystonic movement disorder primarily affecting his lower limbs as well as a sensorimotor neuropathy. Ocular abnormalities include nystagmus, astigmatism and retinal dystrophy causing severe sight impairment. Similarly to his sister, cardiac investigation reveals abnormal repolarisation which is under ongoing review. In addition, there is evidence of postural scoliosis and pronounced lordosis. Prophylactic antimicrobial treatment has been successful in managing a history of recurrent respiratory tract infections. An isolated prolonged APTT and mild transaminitis were found at approximately 3 years of age. MRI brain has shown mal-rotation of hippocampus, retro- cerebellar cyst, thin cervical cord, immature myelin and small basal ganglia small (lentiform nucleus).

**Family 4:**

3 siblings from family 4 are born to healthy consanguineous parents of Pakistani origin. **Patient 4-1** was born at term by assisted delivery following a normal pregnancy. Her developmental milestones were delayed from infancy. At the age of 14 years, there is persistent speech delay as well as difficulty with gross and fine motor skills, and a mildly ataxic gait. Her memory is very good, however there are severe learning difficulties with an inability to read and write, and she does occasionally experience mood swings. She is independent with self-care skills**.** Ocular signs include nystagmus and retinal dystrophy. There is mild skin involvement in the form of eczema which is managed with emollients. Subjectively there is evidence some spinal curvature with a possibility of evolving scoliosis. At present her menstrual cycle is regular.

**Patient 4-2** was born by planned caesarean section, and delivered in good condition. Her phenotype is mild in comparison to her siblings at the current age of 8 years. She suffers from retinal dystrophy and nystagmus. Her learning difficulties are mild and she reached her developmental milestones at the appropriate ages. She is currently pre-pubertal and not reached menarche. She does suffer from anxiety and her skin is mildly dry.

**Patient 4-3** suffers a more severe phenotype in comparison to his siblings. He was also delivered by elective caesarean section and was born in good condition, however global developmental delay was evident from early infancy. At 5 years of age he remains non mobile, with significant hypotonia, as well as dystonia requiring botulinum toxin injections to the lower limbs. He is able to smile but cannot verbalise. Feeding difficulties with an uncoordinated swallow was identified early in infancy and he is now exclusively gastrostomy fed. He suffers from scoliosis, joint laxity and bilateral dislocated hips. There is premature loss of dentition, and bruxism. Like his siblings he also suffers from nystagmus, retinal dystrophy as well as optic nerve hypoplasia. There are no reported cutaneous features. MRI brain reveals small cerebral white matter volume, small cerebellum and pons, delayed myelination, small basal ganglia (lentiform nucleus), small optic nerves and malrotation of hippocampus. There is a history of recurrently lower respiratory tract infections for which prophylactic antibiotics are required. Liver enzymes are found to be abnormally elevated.

**Family 5:**

Siblings in family 5 are from Emirati origin, with a history of multiple consanguinity. These siblings were initially reported by Al-Gazali et. Al 2008.^6^ Patient **5-1** was born weighing 2.2kg following normal pregnancy. In the neonatal period she was diagnosed with transposition of the great arteries and was operated on in the first months of life. There as evidence of developmental delay in the first 2 years of life and microcephaly. She was diagnosed with a significant left divergent squint and bilateral optic nerve coloboma. Skin lesions included a widespread itchy rash with scaling, hyperpigmentation and lichenification. Skin biopsy showed hyperkeratosis and parakeratosis with keratin plug formation. She has required endocrinology follow up due to a variant of septo-optic dysplasia with a finding of a small anterior pituitary on MRI brain. Abdominal scans have revealed a right duplex kidney.

**Patient 5-2** was born by normal delivery weighing 2.79kg following an uncomplicated pregnancy. There was a soft systolic murmur diagnosed as a secundum atrial septal defect on echocardiogram after birth. Ophthalmological examination revealed bilateral iris colobomas and inferior chorioretinal colobomas. He has moderate developmental delay, microcephaly and hypotonia. MRI brain revealed cerebellar vermis hypoplasia, bilateral severe frontal microgyria and delayed myelination. Similarly to his sister there was a small anterior pituitary gland. Skin changes were in keeping with ichthyosis which was confirmed on biopsy. Liver enzymes are elevated.
