# Supplementary material for: SRD5A3-CDG: Emerging Phenotypic Features of an Ultrarare CDG Subtype
Source: Front Genet. 2021 Dec 1;12:737094. doi: 10.3389/fgene.2021.737094 (PMC8671882; doi:10.3389/fgene.2021.737094)

**Figure 3: Colour fundus, autofluorescence, infrared, and OCT images in 3 individuals of 2 families with *SRD5A3*-CDG.**

(A) Optos image of eye with nystagmus (leading to some distortion of the image) - demonstrating mild vascular attenuation, myopic oval disc morphology, and subtle macula reflex abnormalities. The peripheral retina does not reveal any pigmentary abnormalities; (B) delineating 'watershed' zone between relatively preserved central retina, and dystrophic periphery; (C) loss of ellipsoid outside the perifoveal region in both eyes; (E) myopic discs, retinal nerve fibre layer reflexes constrained to macula, Subtle vascular attenuation, no significant retinal pigmentation migration suggestive of retinal dystrophy; (D) myopic discs, retinal nerve fibre layer reflexes constrained to macula, subtle vascular attenuation, no significant retinal pigmentation migration suggestive of retinal dystrophy. (E) abnormal macula with hyper-autofluorescent ring around fovea; (F) loss of ellipsoid - the outer retinal layers beyond the fovea (as highlighted by arrows). (G) Colour image of left eye; (H) hyper-autofluorescent ring illustrating watershed area between preserved central macula function and dystrophic retinal periphery; (I) loss of ellipsoid layer outside the perifoveal retina.

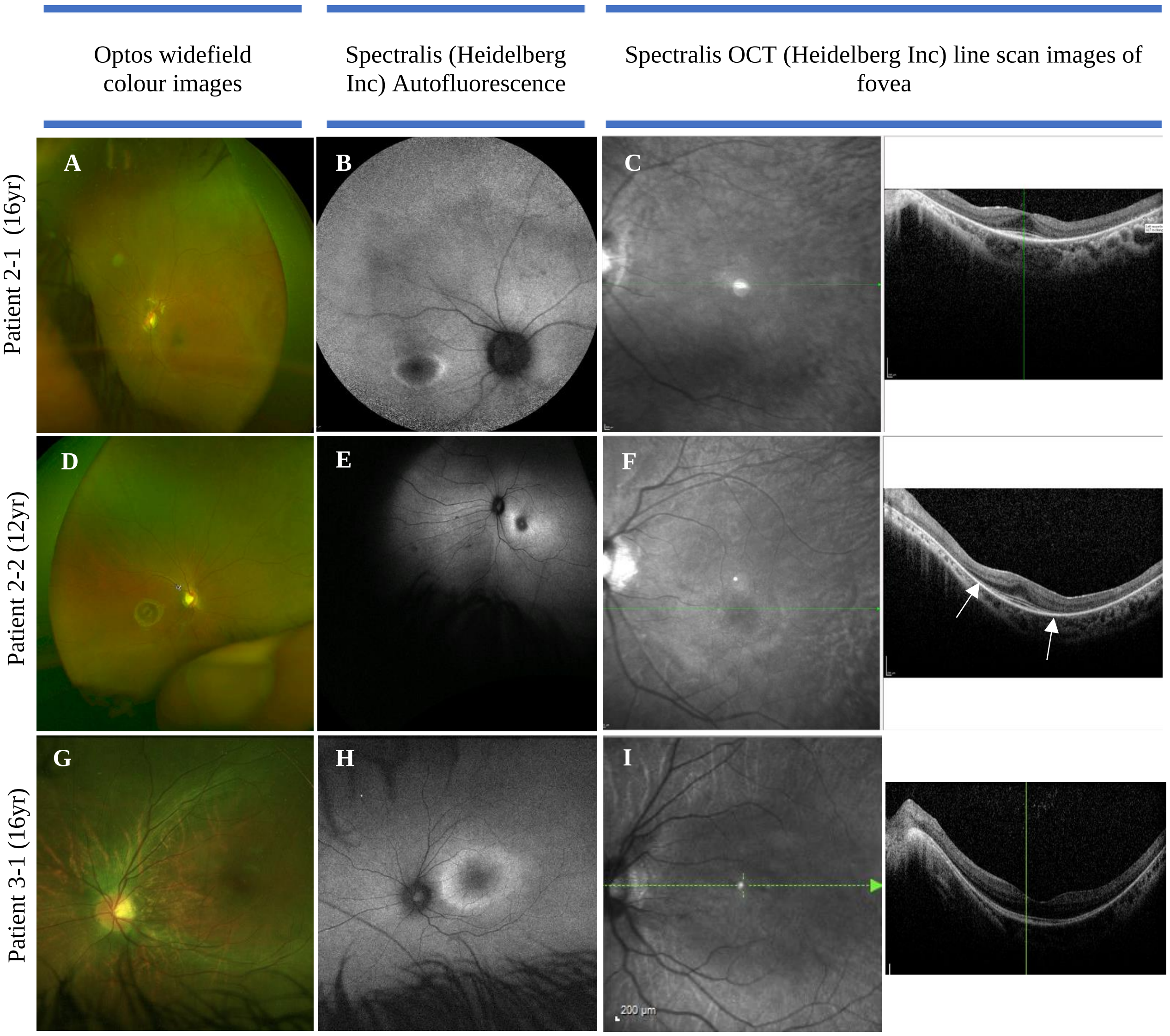

Supplement: Supplementary file 4 [file DataSheet3.pdf]
