# Supplementary material for: SRD5A3-CDG: Emerging Phenotypic Features of an Ultrarare CDG Subtype
Source: Front Genet. 2021 Dec 1;12:737094. doi: 10.3389/fgene.2021.737094 (PMC8671882; doi:10.3389/fgene.2021.737094)

Figure 1: Frequency of symptoms affecting our SRD5A3-CDG cohort
This bar chart demonstrates the number of patients affected by each clinical feature / organ involvement.
Learning difficulties (11/11), ophthalmological signs (11/11), hypotonia (10/11), speech delay (9/11), motor delay (9/11), cutaneous (7/11), scoliosis (6/11), anxiety (5/11), ataxia (5/11), gastrointestinal (5/11), cardiac (4/11), endocrine (4/11), autistic features (3/11), joint laxity (3/11), dystonia (2/11), dysmorphism (2/11), renal (1/11)

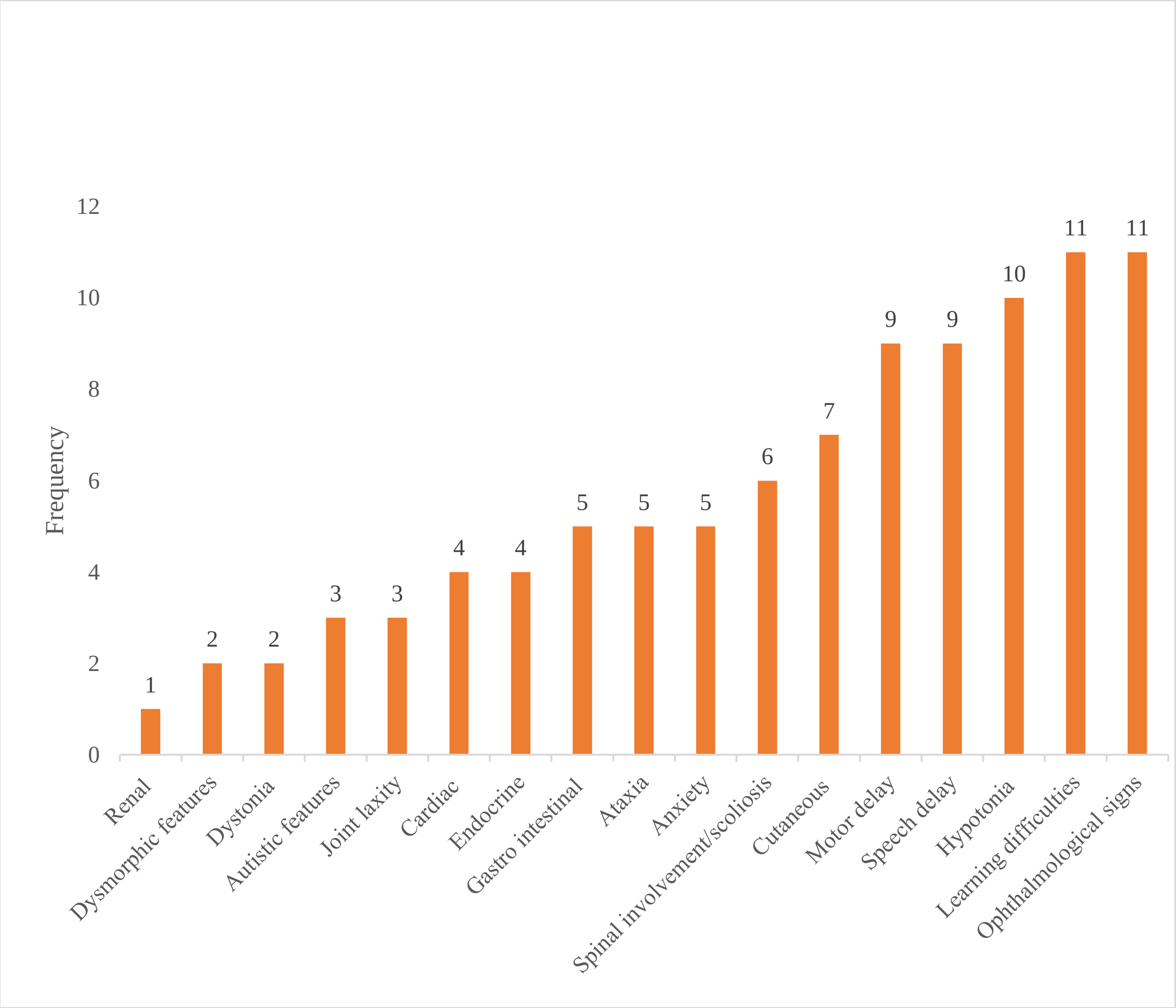

Supplement: Supplementary file 5 [file DataSheet1.pdf]
