# Supplementary material for: SRD5A3-CDG: Emerging Phenotypic Features of an Ultrarare CDG Subtype
Source: Front Genet. 2021 Dec 1;12:737094. doi: 10.3389/fgene.2021.737094 (PMC8671882; doi:10.3389/fgene.2021.737094)
Supplement: Supplementary file 6 [file DataSheet5.pdf]

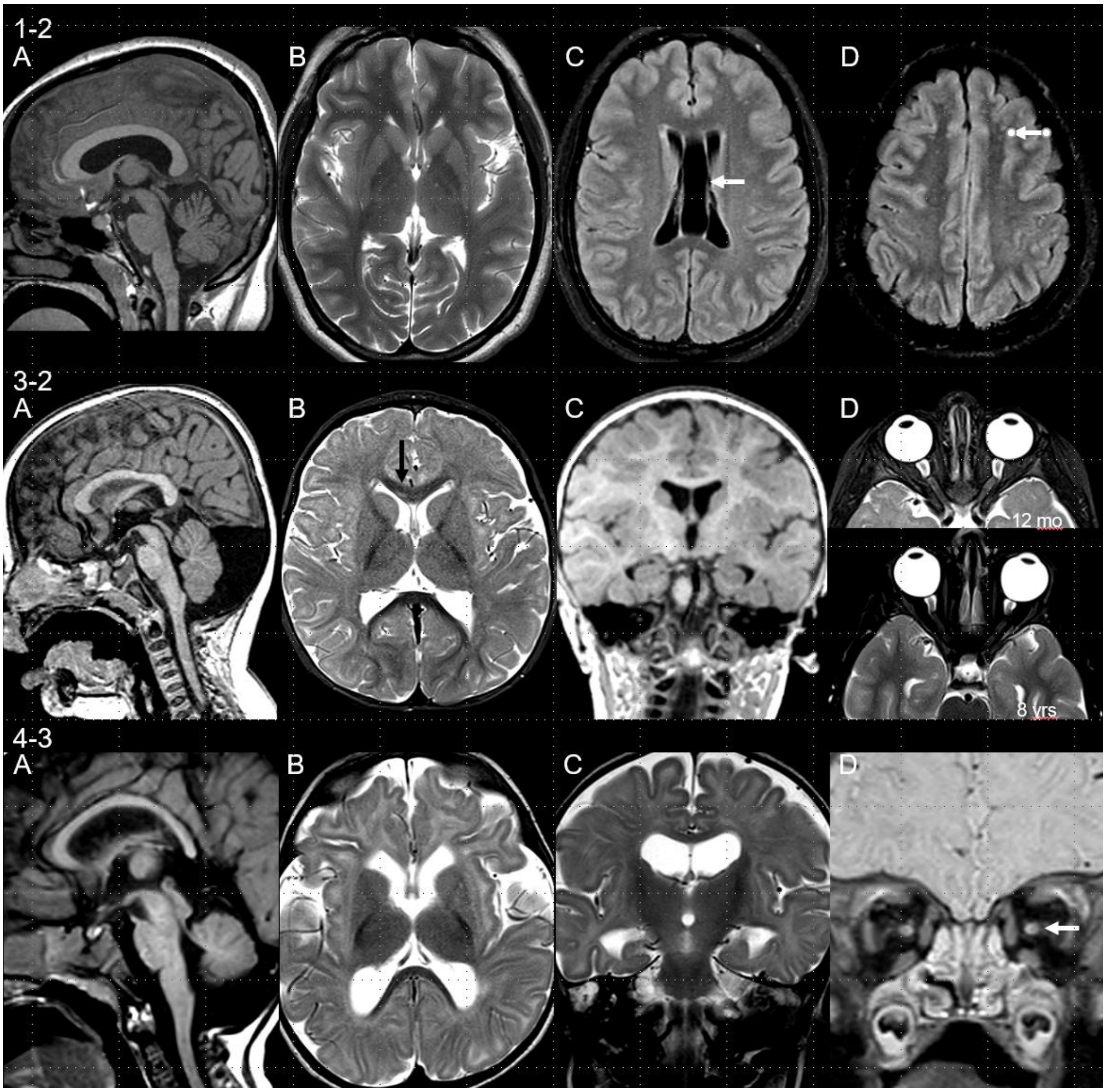

Figure 5: Neuroimaging findings in 3 unrelated patients.

MRI of patient 1-2 at age 15 years shows A) thick corpus callosum and mild cerebellar hypoplasia, B) small basal ganglia, C) cavum septum pellucidum et vergae (arrow) and D) non-specific punctate white matter lesions on fluid-attenuated inversion recovery (FLAIR) sequences (arrow). Patient 3-2 scanned at 12 months of age shows A) retrocerebellar cyst and thin cervical cord, B) signal change of the genu of corpus callosum (immature myelin, black arrow) and small lentiform nucleus, C) malrotation of hippocampi and D) asymmetry of the globes, more prominent on follow-up (lower image). Patient 4-3 at 7 months of age shows A) small cerebellum and pons and drooping splenium, B) microcephaly with volume loss of the white matter, delayed myelination and volume loss of the lentiform nucleus, C) malrotation of hippocampi and D) volume loss of the optic nerves.
